# In Silico Characterization of Toxin-Antitoxin Systems in *Campylobacter* Isolates Recovered from Food Sources and Sporadic Human Illness

**DOI:** 10.3390/genes12010072

**Published:** 2021-01-07

**Authors:** Bishoy Wadie, Mohamed A. Abdel-Fattah, Alshymaa Yousef, Shaimaa F. Mouftah, Mohamed Elhadidy, Tamer Z. Salem

**Affiliations:** 1 Biomedical Sciences Program, University of Science and Technology, Zewail City of Science and Technology, October Gardens, 6th of October City, Giza 12578, Egypt; bwadie@zewailcity.edu.eg (B.W.); g-shassan@zewailcity.edu.eg (A.Y.); sfmouftah@zewailcity.edu.eg (S.F.M.); 2 Department of Agricultural Biotechnology, Faculty of Agriculture, Ain Shams University, Cairo 11566, Egypt; m_abdelfatah@agr.asu.edu.eg; 3 Department of Bacteriology, Mycology, and Immunology, Faculty of Veterinary Medicine, Mansoura University, Mansoura 35516, Egypt; 4 Department of Microbial Genetics, AGERI, ARC, Giza 12619, Egypt

**Keywords:** toxin, antitoxin, *Campylobacter*, MLST, synteny, in silico, genome, domain

## Abstract

*Campylobacter* spp. represents the most common cause of gastroenteritis worldwide with the potential to cause serious sequelae. The ability of *Campylobacter* to survive stressful environmental conditions has been directly linked with food-borne illness. Toxin-antitoxin (TA) modules play an important role as defense systems against antimicrobial agents and are considered an invaluable strategy harnessed by bacterial pathogens to survive in stressful environments. Although TA modules have been extensively studied in model organisms such as *Escherichia coli K12*, the TA landscape in *Campylobacter* remains largely unexplored. Therefore, in this study, a comprehensive in silico screen of 111 *Campylobacter* (90 *C.* *jejuni* and 21 *C.* *coli*) isolates recovered from different food and clinical sources was performed. We identified 10 type II TA systems belonging to four TA families predicted in *Campylobacter* genomes. Furthermore, there was a significant association between the clonal population structure and distribution of TA modules; more specifically, most (12/13) of the *Campylobacter* isolates belonging to ST-21 isolates possess *HicB-HicA* TA modules. Finally, we observed a high degree of shared synteny among isolates bearing certain TA systems or even coexisting pairs of TA systems. Collectively, these findings provide useful insights about the distribution of TA modules in a heterogeneous pool of *Campylobacter* isolates from different sources, thus developing a better understanding regarding the mechanisms by which these pathogens survive stressful environmental conditions, which will further aid in the future designing of more targeted antimicrobials.

## 1. Introduction

*Campylobacter* is considered the most common bacterial pathogen responsible for human gastroenteritis worldwide [1]. Food-producing animals represent the main reservoir for *Campylobacter* infection, and transmission was previously reported to occur through the consumption of food of animal origins, including undercooked chicken, unpasteurized/incomplete milk, and dairy products, and through contaminated drinking water [2].

Several studies have focused on the identification and characterization of virulence factors in *Campylobacter* through which infection is mediated. These factors are primarily associated with the expression of genes involved in colonization, cell invasion, motility, and toxin production [3,4]. Such investigations have provided the research community with a better understanding to identify potential drug targets and to develop therapeutic interventions. However, the prevalence of cases with campylobacteriosis is still on the rise, albeit with the availability of a wide variety of antimicrobial agents. Despite the considerable importance of dissecting the mechanisms by which the virulence factors drive the infectivity of *Campylobacter*, our understanding of how *Campylobacter* is defending itself against antimicrobials is still lacking.

Bacterial cells are continuously exposed to exogenous genetic elements by means of horizontal gene transfer and phage infections that put them at risk of cell death. Generally, plasmids are transferred to other bacterial cells either by transformation or conjugation, and while it is commonly known that plasmids confer a selective advantage to the recipient bacterial cells, such as antibiotic resistance, other plasmids (e.g., Col plasmids) might contain bacteriocins that are proteins that kill other bacterial cells [5]. On the other hand, phages are well-known for their ability to infect and eventually lyse the bacterial cells. To be protected against such hazards, bacterial cells utilize three general defense strategies: (1) preventing pathogen entry, (2) adopting abortive infection (suicide/dormancy when infected), and (3) utilizing cellular immunity, which encompasses innate mechanisms (such as restriction modification systems) and adaptive mechanisms (such as CRISPR-Cas systems) [6]. In response to such defense systems, phages have, in turn, developed ways to counteract these systems, leading to an evolutionary arms race influenced by the extensive coevolution of both phage and host [7]. Since some defense systems are antagonized by phages, bacterial genomes encode multiple defense systems in discrete genomic loci called “defense islands”; these sites are frequently found in sites undergoing horizontal gene transfer, which explains the rapid diversification of these systems [6]. One of these defense systems is the toxin-antitoxin (TA) system, which is the primary focus of this study.

Toxin-antitoxin (TA) modules are comprised of a stable toxin that inhibits cellular growth and an unstable antitoxin that counteracts its action. TA modules can be generally classified into four major types (types I, II, III, and IV) based on the mechanism through which the antitoxin neutralizes the activity of the toxin protein, in addition to two minor types (types V and VI). Type I and type III antitoxins are RNA molecules that regulate the activity of their toxin counterparts through inhibiting the translation of toxin mRNA or directly inhibiting the toxin protein, respectively. Type II antitoxins are proteins that directly bind and inhibit toxin proteins, unlike type IV antitoxin proteins that neutralize the toxin’s activity without direct interaction [8]. Unlike types I–IV, types V and VI antitoxins contribute to the degradation of their toxin counterparts, either by acting as an RNase leading to the cleavage of toxin mRNA or forming a complex with a protease to cleave the toxin protein, respectively [9,10]. TA systems were first discovered on plasmids, and since then, they have been labeled as mobile genetic elements that are frequently transferred horizontally [8,11]. All types of TA modules are also enriched on bacterial chromosomes; however, the composition and quantities of chromosomal TA modules are highly variable among different bacterial species and even between strains [12,13]. However, no clear distinctions can be drawn between TA modules and the biology of a given species. Nonetheless, these TA systems are more frequently found in organisms living in hostile environments with recurrent events of horizontal gene transfer [12,13]. Toxins have been shown to utilize a myriad of molecular mechanisms to inhibit cell growth, most of which are well-characterized [8,14,15]. On the contrary, the biological functions of most TA modules remain elusive. Some functions have been proposed, however; the three most common ones include postsegregational killing (PSK) aimed at the stabilization of mobile genetic elements, abortive infection through altruistic suicide, and dormancy through which bacterial cells remains dormant and become antibiotic-tolerant [8,16].

As previously mentioned, the composition and quantities of TA modules are highly variable among organisms. Most studies are biased towards the characterization of TA modules in model organisms such as *Escherichia coli K12*. Nonetheless, little is known about the composition and numbers of TA modules in *Campylobacter*. In this study, we performed a comprehensive in silico screen for TA modules on 111 *Campylobacter* isolates sampled from different sources and assessed the distribution and conservation of the predicted TA modules among those isolates.

## 2. Materials and Methods

### 2.1. Bacterial Isolates and Culture Conditions

A total of 111 *Campylobacter* isolates (90 *C. jejuni* and 21 *C. coli*), collected between 2017 and 2018 in Cairo, Egypt, were isolated from foods of animal origin (commercial broiler carcasses (*n* = 30), milk and unpasteurized/incompletely pasteurized dairy products (*n* = 24)) and fecal clinical samples from patients suffering from diarrheal illness (*n* = 57) admitted to two different hospitals in Cairo, Egypt (Supplementary File S1). A stratified randomized sampling was conducted to collect food samples from different retail stores located around the study region. Isolation and enumeration of *Campylobacter* isolates from various food matrices was achieved according to the ISO 10272-1 (enrichment method; detection of *Campylobacter* spp. after selective enrichment). Stool samples from human patients were grown on modified charcoal cefoperazone deoxycholate agar (mCCDA). Plates were then incubated for 48 h at 42 °C under anaerobic conditions using AnaeroGen™ 2.5-L sachets (Oxoid, Basingstoke, UK). Confirmation of *Campylobacter* at the genus level was performed using PCR identification of the *16S rRNA* gene [17]. Confirmation of *C. jejuni* and *C. coli* at the species level was performed by PCR detection of the *mapA* gene and *ceuE* gene [18,19], respectively. All isolates were sub-cultured from −80 °C frozen stocks onto Mueller-Hinton (MH) agar (Oxoid, Basingstoke, UK).

### 2.2. Multilocus Sequence Typing (MLST)

Sequence types (ST) and clonal complexes (CC) of all isolates were determined from Whole Genome Sequencing (WGS) data and queried against the sequences in the BIGSdb database [20] which automatically identifies alleles and assigns sequence types (ST) and a clonal complex (CC) for each isolate sequence.

### 2.3. Data Acquisition

*Campylobacter* genome assemblies (FASTA) for each corresponding isolate record were retrieved from the BIGSdb database (https://www.ncbi.nlm.nih.gov/bioproject/PRJNA576513). BIGSdb is an open-source web-based platform that integrates phenotype and sequence data for a multitude of bacterial species building on multilocus sequence typing (MLST) data [20].

### 2.4. Genome Annotation

Since the bacterial species for all the isolates identified are either *C. jejuni* or *C. coli*, genome annotation was performed against different strains for the above species. Contigs from BIGSdb (FASTA files) for each isolate were annotated using prokka v1.14.5 against a custom database [21]. The custom database was built using annotated genomes for different *C. jejuni* and *C. coli* based on the TADB (Toxin-Antitoxin database) and Microbesonline list of available strains [22,23]. A list of *Campylobacter* reference genomes can be found in Supplementary File S2. Annotated genomes (GBK files) were downloaded from Microbesonline (http://www.microbesonline.org/) and NCBI (https://www.ncbi.nlm.nih.gov/pmc/articles/PMC3570591/) and were converted to protein format (FAA files) using in-house python scripts. Then, all protein sequences were concatenated, clustered, and added to prokka’s genus directory to be used as a reference for annotation. The remaining parameters were set to default. For each isolate sequence, GFF and FNA output files were stored and used for downstream analyses.

### 2.5. In Silico Screening for TA Systems

To identify toxin-antitoxin genes in each isolate, TADB and PADS Arsenal (prokaryotic defense system-related genes) databases were used, which contain both predicted and experimentally validated TA sequences. TADB provides ample data about bacterial type II TA systems covering 6193 loci, 105 of which are experimentally validated [22]. PADS Arsenal is a public database integrating 18 different categories of defense systems (including TA systems), with a total of 6,600,624 annotated genes from 63,701 genomes covering 33,390 species of bacteria and archaea [24]. FASTA files of toxin and antitoxin genes were downloaded from TADB and PADS Arsenal databases. FNA output files for each isolate were aligned against TA genes from both TADB and PADS Arsenal using the BLSATN command line tool to identify TA genes present in each isolate [25]. All csv outputs for each database were merged and records having e-values > 10^−5^ were filtered out. In addition, only reference genome assemblies with a level of assembly labeled as “Complete” or “Chromosome” were included, as opposed to “Contig” or “Scaffold” from PADS Arsenal hits. Finally, in order to account for gene coverage, the percentage identity was multiplied by the alignment length followed by scaling values from 0 to 1 using “min–max normalization”. The scaled score represents the quality of the alignment given that multiple e-values were equal to zero because of the relatively narrow search space. It is worth noting that only four out of 111 *Campylobacter* isolates harbored type I antitoxin genes with no toxin counterparts, 3 of which are *Campylobacter jejuni* (7672, 7673, and 7690) and 1 *Campylobacter coli* (7700). Interestingly, these genes represent experimentally validated antitoxin genes according to TADB. An annotated file for all TA modules from TADB and PADS Arsenal can be found in Supplementary File S3.

### 2.6. Protein Domain Analysis

The protein domain for each toxin-antitoxin gene was identified using the NCBI conserved domain database (CDD) [26] and InterProScan web-based search tool [27]. These tools were used to confirm that the identified TA genes bear a TA-related functional domain.

### 2.7. Sequence Alignment and Clustering

In order to evaluate the sequence conservation of TA-related domains among hits from different genome assemblies, multiple sequence alignment was implemented for each locus tag possessing a TA-related domain. Sequence alignment was performed using the msa R package using default parameters [28]. In addition, hierarchical sequence clustering was performed using the seqinr R package [29] for all locus tags regardless of having a domain for visualization purposes.

### 2.8. Synteny Analysis

Syntenic regions between isolate genomes were identified to better assess the degree of conservation of homologous genes and gene order among isolates containing TA systems. Accordingly, for each isolate, only the contigs containing at least one TA system were selected for synteny analysis. Matching syntenic regions between two contigs are accomplished by reciprocal blast implemented in the DECIPHER R package using k-mer exact matching, which is identified as a syntenic hit [30]. Pairwise analysis was conducted on 64 contigs from 54 isolates containing at least one TA system; for each contig pair, the number of syntenic hits, number of syntenic blocks, and percentage coverage of contig length were recorded. Hits coverage was calculated as:

$$\frac{\sum_{k}^{n}W_{k}}{\min\left(l_{i},l_{j}\right)}$$

where *W_k_* corresponds to width of syntenic hit *k*, *n* is the total number of syntenic hits, and *l_i_* and *l_j_* correspond to the length of contigs *i* and *j*, respectively. The hits coverage was used to draw a clustered heatmap to represent the degree of overlap between the corresponding contigs.

### 2.9. Protein–Protein Interaction (PPI) and Functional Enrichment Analysis

A protein–protein interaction network was constructed using STRINGdb with an interaction score cutoff of 0.9 (highest confidence), and a second shell of no more than five interactors was added [31]. For each cluster of the PPI network, a GO (gene ontology) enrichment analysis was performed on the genes comprising each cluster for “Biological Process” and “Molecular Function” ontologies using the enrichGO function with default parameter, which is implemented in the R package clusterProfiler [32].

### 2.10. Statistical Analysis

All statistical tests were performed using R version 4.0.0 (2020-04-24) [33]. *p*-value < 0.05 were considered significant.

## 3. Results

### 3.1. Distribution of TA Systems across Campylobacter Isolates

**Figure 1:**
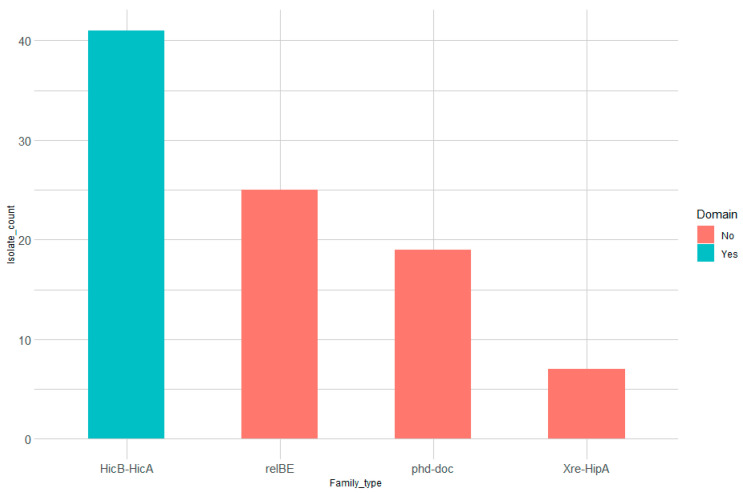
Isolate count per toxin-antitoxin (TA) family type. Bar lengths correspond to the number of isolates containing TA systems belonging to a given TA family. Blue bars represent TA systems where both toxin and antitoxin genes harbor a TA-related domain, while red bars represent TA systems where either the toxin or antitoxin gene harbor a TA-related domain.

The diversity of toxin-antitoxin within and between isolates allowed us to better dissect their distribution based on their structural domain and reference genome. Therefore, we performed extensive screening using TADB and PADS Arsenal databases as references and only considered aligned sequences with >60% coverage. After removing false positives due to multiple matches with the same sequence from different genome assemblies or same genomic regions in each isolate mapping to different sequences, we obtained a total of 12 toxin genes and 14 antitoxin genes (Figure S1) across 66 out of 111 isolates. From these isolates, 54 isolates possess at least one TA system, and 27 isolates possess structurally validated TA systems. According to TADB, the predicted TA systems belong to four type II TA families (Figure 1). Furthermore, the alignment quality for each toxin-antitoxin across the isolates was assessed based on a scaled score that takes into account the alignment coverage (see Methods). Most of the toxin-antitoxin genes (grouped by domain) had alignment scores > 95% (Figure S2) reflecting a high similarity between the isolate sequences and their corresponding genes from various genome assemblies. All of the toxin/antitoxin genes discussed hereafter belong to type II TA systems; however, we also obtained hits for *cjrA-RNA* (a type I antitoxin found in pVir plasmid identified in *C. jejuni* 81-176) in four isolates (Supplementary File S4) from the TADB database. Interestingly, this antitoxin was the only experimentally validated hit from TADB [34], but since we could not find its associated toxin, we decided not to perform further downstream analyses on *cjrA-RNA*.

**Figure 2:**
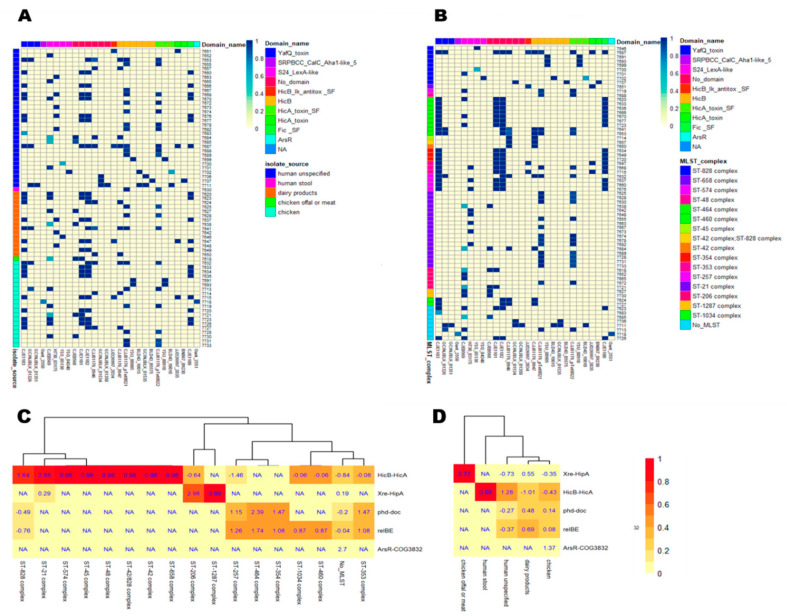
Heatmap showing the distribution of TA genes and families across isolates, isolate sources, and clonal complexes. In (**A**,**B**), each cell represents the occurrence of a TA gene (represented by the locus tag shown on the x-axis) for each isolate (shown on the y-axis). For the identification of a hit, a cut-off threshold of alignment length > 60% was applied, as proposed in [36]. The color conventions show the scaled score representing the alignment quality (see Methods). TA genes are grouped by their protein domain (shown as a colored track on top). In (**C**,**D**), each cell is colored by a normalized score, where the number of isolates in a given isolate source/clonal complex and a given family type is divided by the total number of isolates in a given isolate source/clonal complex, regardless of family type. In addition, cells are labeled by their standardized residuals as calculated from Pearson’s chi-square test. Residuals higher or lower than 2 or −2, respectively, show a statistically significant association (*p* < 0.05). (**A**) Isolates are grouped based on the isolate source (represented by a colored track on the left). (**B**) Isolates are grouped based on their clonal complex (represented by a colored track on the left). (**C**) Hierarchically clustered heat map between the TA family type and clonal complex. (**D**) Hierarchically clustered heat map between the TA family type and isolate source.

Next, the distribution of toxin-antitoxin genes across isolates as a function of the isolate source and clonal complex was investigated. We wanted to check if isolates that are sampled from the same animal/clinical source or share the same clonal complex also share the same toxin-antitoxin genes. The distribution of TA genes was found to be highly sparse as a function of the isolate source (Figure 2A). On the other hand, the same distribution appears to be less sparse as a function of the clonal complex (Figure 2B). To further investigate whether the isolate source or clonal complex are dependent on the presence of different TA families, a Pearson chi-square test of independence between each clonal complex/isolate source and family pair were performed. According to both the chi-square and Fisher test, no significant association between the isolate source and family type was found, suggesting that the isolate source and TA family type are independent of each other. In contrast, there was a strong association between four CCs and three TA family types confirmed through Fisher’s exact test (Supplementary File S5), suggesting a dependent relationship between some TA family types and certain clonal complexes (Figure 2D).

**Figure 3:**
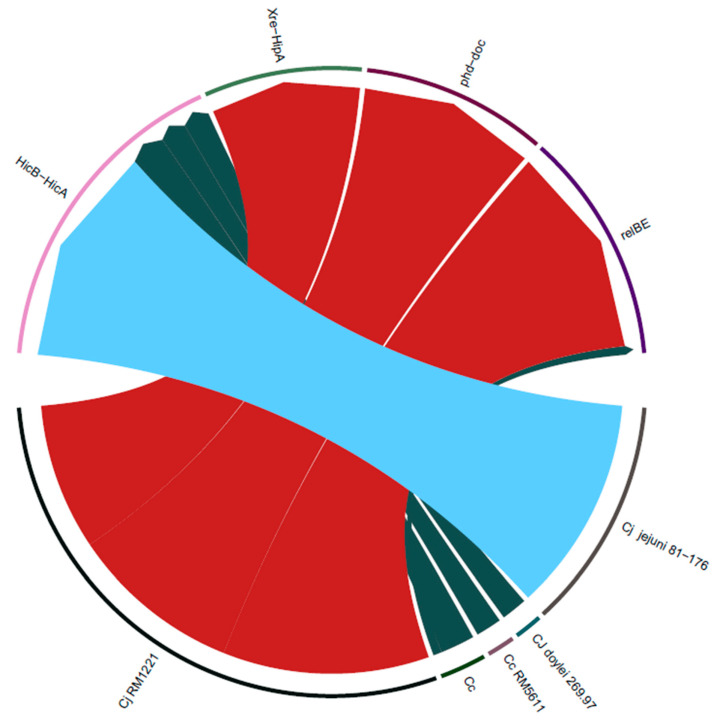
Circos plot showing the distribution of type II TA families from different strains. The lower nodes correspond to different genomic strains from which arcs are drawn towards the corresponding TA families in the upper nodes. The thickness of each arc corresponds to the number of isolates containing TA systems that belong to a given TA family and are matched from a given strain. C.j, *Campylobacter jejuni* and C.c, *Campylobacter coli.*

Since most of the identified TA systems are based on BLAST alignment against the TADB and PADS databases, and since contigs were assembled in isolates from different veterinary/clinical sources, it is highly plausible that the matched TA systems are found in different strains. Accordingly, we wanted to connect each TA system identified in each isolate to the genome where it was identified (Figures S3 and S4). In addition, we also checked the association between TA family types and the genomic strains where they were identified (Figure 3). Interestingly, we observed that most of *HicB-HicA* TA systems were identified from the *C. jejuni 81-176* strain, while almost all the other families were identified from *C. jejuni RM1221* (Figure S3). It is also worth noting that the *HicB-HicA* family was the only family identified that harbors a TA-related domain. (Figure 1). On the other hand, either the toxin or antitoxin in the other families contain a TA-related domain. This pattern can be seen in Figure S4, where only the TA systems that contain both the toxin and antitoxin domain are considered and showed the absence of connections from *C. jejuni RM1221.*

### 3.2. Sequence Alignment and Clustering

**Figure 4:**
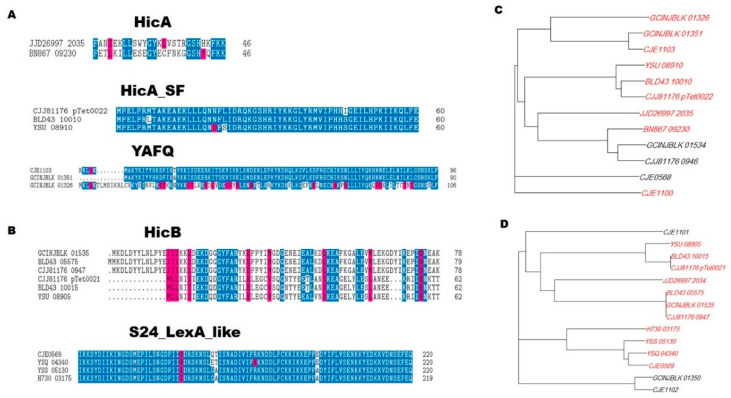
Multiple sequence alignment of locus tags for each domain. (**A**) Sequence alignment of toxin genes for each identified domain. (**B**) Sequence alignment of antitoxin genes for each identified domain. (**C**) Dendogram representing sequence clustering of all toxin genes, locus tags highlighted in red contain toxin domains. (**D**) Dendogram representing sequence clustering of all antitoxin genes, locus tags highlighted in red contain antitoxin domains. Residues highlighted in blue are ≥50% conserved, and those highlighted in pink show similar residues.

Given the variety of genome assemblies from which the TA genes were identified and the heterogeneity of the assembled isolate genomes, we investigated the degree of similarity between the toxin and antitoxin genes from different genome assemblies representing the bacterial strains. Multiple sequence alignments were performed between toxin/antitoxin genes for each given domain, as well as those with no reported domain (Figure 4). Some of the domains (HicA_SF, YAFQ, and S24-LexA-like) showed >95% similarity between their constituent genes, while other domains such as *HicA* and *HicB* showed relatively low similarity. Despite the low similarity, those genes had highly conserved sites, as shown in *HicB* (Figure 4B), and their e-values, as reported by the CDD, with at least 10^−3^ and 10^−8^ for genes designated to harbor the *HicA* and *HicB* domains, respectively, which explains why these genes were assigned to their respective domains. Finally, we clustered all the locus tags by their sequence similarity to show the hierarchical relationship between the locus tags from different genome assemblies (Figure 4 C,D).

### 3.3. Synteny Analysis

**Figure 5:**
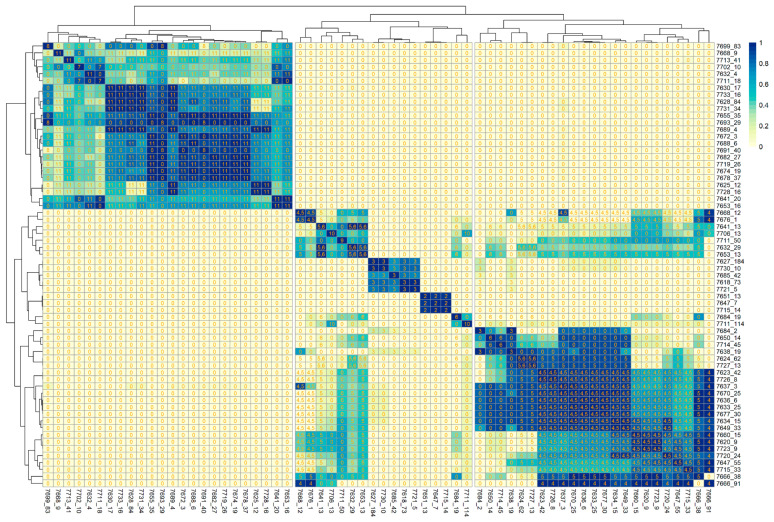

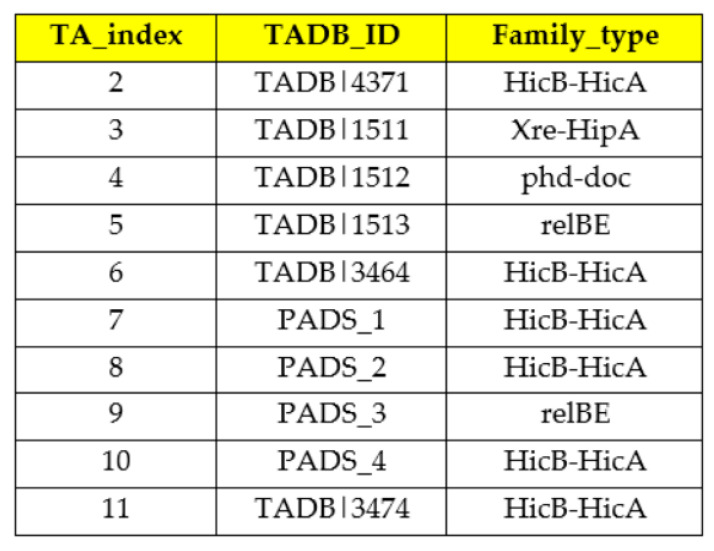
Clustered heatmap mapping the coverage of syntentic hits between each contig. Each cell is labeled by an index indicating the TA systems shared by each contig pair (table above). The color conventions show the coverage proportion between each contig pair; 1 means 100% coverage, and 0 indicates no synteny between corresponding contigs. The columns and row labels represent the isolate id, along with the contig number.

In this study, we sought to characterize the TA systems in a diversified set of *Campylobacter* isolates with a sparse genomic organization. Hence, it is crucial to assess the homology and the degree of conservation between the isolates in terms of their constituent TA systems. Additionally, despite the fact that most TA systems are scattered in different regions across the genome, the synteny between those regions or so-called blocks between different isolates should be conserved. Therefore, we quantified the syntenic blocks between all pairs of contigs that are known to contain a TA system; we restricted the analysis only to contigs containing TA systems to increase the signal-to-noise ratio compared to a whole-genome comparison. The shared synteny between two respective contigs was quantified using the hits coverage, as described above. The contigs were then hierarchically clustered based on the coverage, and the TA systems found in each contig pair were overlaid to show which cluster of contig pairs shared a specific TA system (Figure 5). Some clusters showed high synteny and were significantly enriched in only one TA system, such as those contig pairs containing TADB|4371 (*HicB-HicA*) and TADB|1511 (*Xre-HipA*). Interestingly, there were some clusters containing contig pairs that shared two TA systems in each contig, such as (*phd-doc* and *relBE*) or (*relBE* and *HicB-HicA*), so, in those isolates, those two TA systems could coexist together.

### 3.4. Functional Enrichment and PPI Network

**Figure 6:**
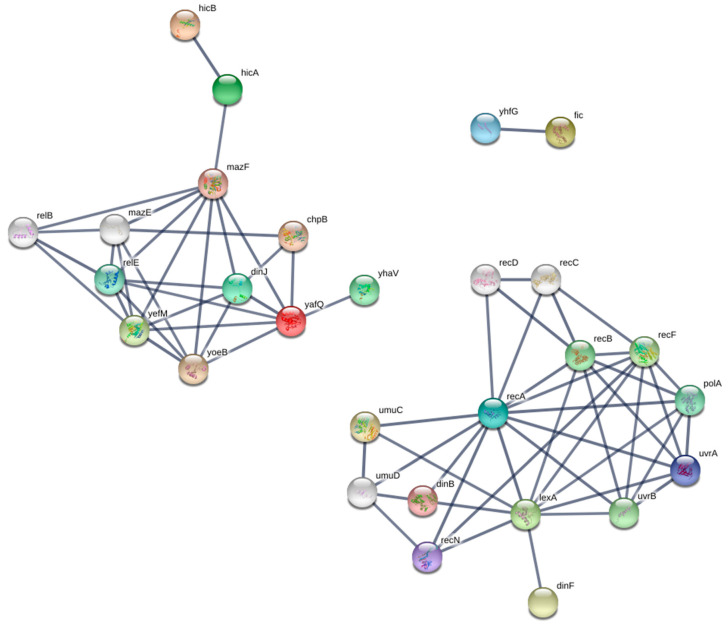
Protein–protein interaction (PPI) network. Each node represents a given protein, and the edges represent the degree of association between the given nodes. The network is divided into 3 independent clusters; the top-left cluster shows the PPIs between the interactors with *yafQ-hicA-hicB* as the seed/query nodes. The bottom-right cluster shows the PPIs between the interactors with *lexA* as the seed/query node. The top-right cluster shows a single interaction involving *fic* as the seed node.

**Figure 7:**
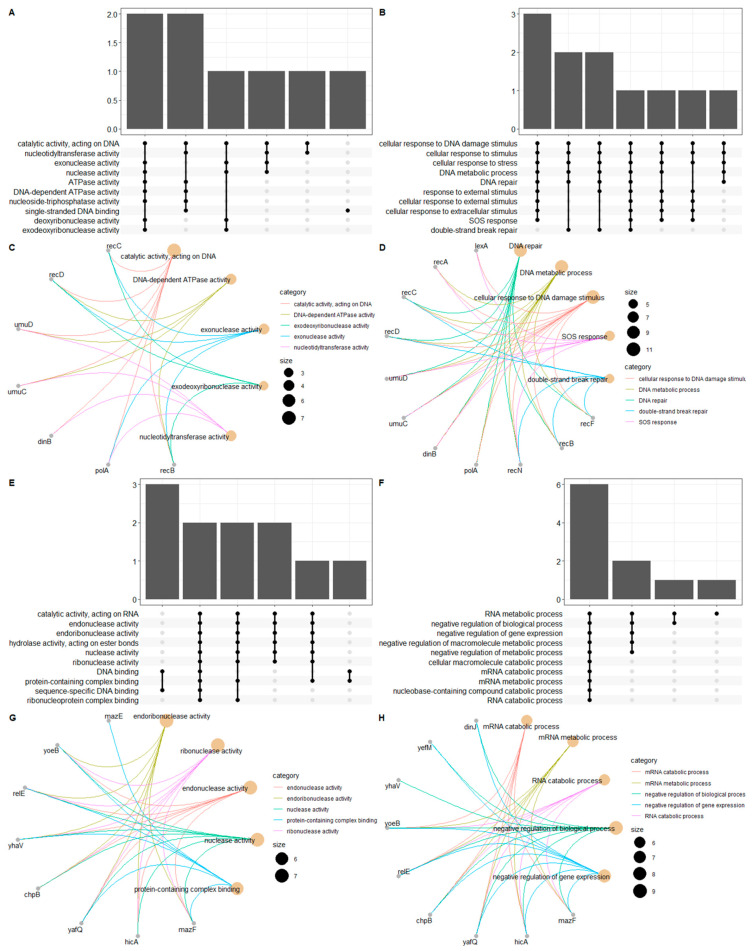
Functional enrichment analysis. (**A**–**D**) Show the enrichment analysis of the cluster involving *lexA* as the seed node, and (**E**–**H**) show the enrichment results of the cluster involving *yafQ-hicA-hicB* as seed nodes (Figure 6). In panels (**A**,**B**,**E**,**F**), upset plots are drawn to visualize the intersection between the gene ontology (GO) terms in a given ontology (horizontal labels). Every possible intersection is represented by the bottom plot, and the frequency (i.e., number of genes) is shown on the top vertical bar plot. In panels (**C**,**D**,**G**,**H**), circular gene-concept networks are drawn to visualize the interaction between each gene and its associated GO term. The edges are colored according to the GO term, and the node size reflects the number of genes associated with each category. Panels (**A**,**C**,**E**,**G**) correspond to enrichment results for GO “molecular function” ontology, while panels (**B**,**D**,**F**,**H**) correspond to enrichment results for GO “biological process” ontology.

In order to comprehensively characterize the identified TA systems, the protein–protein interaction/association between the identified structural domains found in multiple toxin-antitoxin genes and their underlying functional insights were investigated. The resulting network was divided into three main clusters, including the following seed domains (i.e., the ones present in our dataset): (1) *yafQ-hicA-hicB* interactors, (2) *lexA* interactors, and (3) *Fic* interactors (Figure 6). Only *yhfG* was reported to interact with *fic*, and although it is an uncharacterized protein, it is believed that it might be the putative antitoxin for *Fic* [35]. Next, a GO enrichment analysis was performed on the remaining two clusters as a means of exploring the molecular function and biological pathways through which these genes act. Both clusters were enriched for nuclease activities, with the *yafQ* cluster mainly enriched for endonucleases while the *LexA* cluster was enriched in exonucleases and nucleotidyltransferases (Figure 7 A,E). On the other hand, they were not part of the same biological processes, as *LexA* was enriched in DNA repair and the SOS response in contrast to *yafQ*, which was enriched in mRNA metabolism and negative regulation of the gene expression.

## 4. Discussion

In this study, we set out to identify and characterize toxin-antitoxin modules in *Campylobacter* isolates sampled from different clinical/animal sources using in silico screening. We first performed a comprehensive BLAST search against both TADB and PADS arsenal databases to identify previously predicted toxin-antitoxin genes; we then performed a domain analysis to separate between TA modules where both genes contain a structurally validated TA domain and those with only one component possessing a TA-related domain (Figure S1). Most of the TA families identified were mapped to TA modules where either the toxin or antitoxin possess a TA-related domain (*relBE*, *phd-doc*, and *Xre-HipA*) compared to *HicB-HicA* as the only family where both components are structurally validated (Figure 1). All of the identified families are type II TA modules, which contain antitoxins that usually contain an N-terminal DNA-binding domain for transcriptional autoregulation and a C-terminal domain that binds directly to its toxin counterpart [37,38]. *RelBE* is one of the examples where the direct inactivation mediated by the antitoxin occur through the inhibition of catalysis at the toxin’s active site [39]. On the other hand, a defining feature of many toxins is their ability to bind and degrade/cleave their targets enzymatically and, thus, impede the physiological cascades, leading to bacterial growth inhibition. *HicB-HicA* and *RelBE* are both mRNA endoribonucleases that bind to and cleave cellular mRNAs, with the latter being ribosome-dependent and the former ribosome-independent [8]. *HicB-HicA* and *RelBE* modules are also functionally similar to the well-characterized *MazEF* TA modules, especially in *E. coli (strain K-12)*. The toxin *MAZF* is a sequence-specific ribosome-independent mRNA endoribonuclease that recognizes and cleaves a ~7-nt region with a central ACA sequence at the 5′ end of the underlying mRNA, whose secondary structure conformation also influences the recognition and cleavage site [40,41]. The impact of this TA module on the physiology of *E. coli* cells is still debatable; however, it has been suggested that programmed cell death (PCD) is highly dependent on the presence of *MazEF* modules, in addition to quorum-sensing pentapeptide and the extracellular death factor (EDF) [42,43]. However, it has also been reported that *MazEF*-mediated PCD was not reproducible, where the *E. coli* strains (MC4100 and its *MazEF* derivative) failed to show a *RelA* phenotype [44] which demands further scrutiny and skepticism regarding the *MazEF-*mediated PCD hypothesis in future studies. It has also been suggested that both *MazEF*, along with *DinJ-YafQ* TA systems, contribute to biofilm formation [45]. Despite the strong association between *HicB-HicA* and *RelBE* functions to that of *MazEF* TA modules (Figure 6), we cannot conclude that this is indeed the case, especially in *Campylobacter*, because the stress response mediated by *MazEF* has been shown to be strain-dependent in *E. coli* [46], which makes it even more challenging to make inferences on the species level. Unfortunately, there are no/few functional studies concerned with the characterization of the stress response mediated by *HicB-HicA* in *Campylobacter* to reinforce the findings mentioned in this study.

As for the other identified TA families, like *Phd-Doc* (as mentioned in TADB), we observed *Fic* domains in the toxin component belonging to this TA module, and unlike *HicB-HicA*, *Fic* toxins were previously observed in *Campylobacter fetus* [47], and a *Phd-Doc* TA module was recently identified in *C. jejuni YH002* [48]. *Fic* domains function through post-translational modification of their target proteins, specifically by the addition of AMP on key functional residues, thus regulating metabolic functions [49]. Accordingly, activation of the *Fic* toxin can slow cell growth and allow bacterial cells to enter a dormant state [47,50]. These functional cues provide insights about possible mechanisms through which *Campylobacter* species utilize TA modules to survive under different stresses in different environments. The last TA family identified was *Xre/HipA* (as mentioned in TADB), which was observed in seven *Campylobacter* isolates. The antitoxin of this family contained a *S24-LexA*-like domain, which corresponds to the well-known *LexA* transcriptional regulator of the SOS response to DNA damage [51] (Figure 6 and Figure 7). Although it is not considered a putative antitoxin, it was previously reported that some TA systems were regulated by the SOS response, including the *yafNO* TA module in *E. coli* [52]. This was partially explained, because this module lies downstream of the *dinB* gene, which encodes the translesion DNA polymerase IV that is associated with adaptive mutation; the *dinB* gene is also repressed by *LexA* and is part of the *yafNO* operon, which explains why this TA module is regulated by the SOS response [53]. Furthermore, there are also multiple domain architectures where *S24-LexA*-like domains lie downstream of the HTH (helix-turn-helix) *Xre* family, which constitutes a *HipB* antitoxin that lies in the same operon with the *HipA* toxin [54,55]. This might explain why TADB has attributed the *S24-lexA*-like domain to the *Xre-HipA* module. However, it is worth noting that this is just a prediction and that the role of transcriptional regulators like *LexA* in the regulating and determining of the functionality of TA systems is unknown [53].

Next, the overrepresentation of TA systems and families was assessed in relation to the isolate sources and MLST clonal complex (Figure 4). No significant differences between the distributions of TA families were detected among different isolate sources. However, some MLST clonal complexes overrepresented TA families, according to the Fisher exact test. MLST provided invaluable insights into the population genetics of *Campylobacter* species, especially in discerning bacterial relationships, albeit not as discriminatory as pulse-field gel electrophoresis (PFGE) [56]. In addition, it was proven useful to identify sequence types (ST) that fall into clusters called clonal complexes associated with specific niches, which might help in delineating transmission routes for human infection [56]. A significant association between the ST-21 complex and *HicB-HicA* was observed compared to other complexes; more specifically, 12 out of 13 ST-21 isolates harbor a *HicB-HicA* TA module. The ST-21 complex is considered one of the largest clonal complexes found in *Campylobacter* isolates, constituting 26% of all submitted isolates to the pubmlst database [57]. It has also been previously reported that *Campylobacter* isolates belonging to the ST-21 complex are resistant to ciprofloxacin [58], which would be an interesting association to investigate with respect to the presence of *HicB-HicA* TA modules. As per our knowledge, this is the first study reporting the association between TA modules and *Campylobacter* clonal complex, and further investigations are required to validate this premise.

Given the dynamic nature and high mobility of TA modules through recurring events of horizontal gene transfer, it was imperative to evaluate the shared synteny between isolates in terms of the TA modules they harbor, thus quantifying the relatedness of the isolates to each other as a function of the TA systems they share (Figure 5). Interestingly, we observed defined clusters of isolates sharing a certain TA system and even coexisting pairs of TA systems belonging to different families. This type of analysis offers a new perspective to evaluate the relationship between isolates in terms of the TA systems that reside in shared syntenic blocks.

In this study, we only scratched the surface to identify and characterize different TA modules in a fairly large set of *Campylobacter* isolates. However, there are still so many unanswered questions and future interrogations that need to take place. Nathan Fraikin et al. proposed some interesting challenges facing the field of TA systems that currently remain untackled. For example, how genes that are so unpredictably distributed and impermeable to fixations can be central in essential processes such as the stress response. In addition, the mechanisms by which TA systems are transferred between hosts and integrate between genomes remain unresolved [53].

More fundamentally, we still lack basic functional understandings regarding wild-type TA modules. For instance, mRNA targets of TA modules such as HicB-HicA remain largely unknown, as well as the biological processes through which these modules function. Additionally, given the heterogeneous pool of TA modules and their diversity among different bacterial species, our basic understanding about the context-specific cellular response to stress is limited. Indeed, more attention is required to properly characterize and gain mechanistic insights about how these small modules regulate cellular physiology in different environments.

## Supplementary Materials

The following are available online at https://www.mdpi.com/2073-4425/12/1/72/s1: Figure S1: Isolate count per toxin-antitoxin gene. Figure S2: Distribution of alignment score for each toxin-antitoxin domain. Figure S3: Circos plot showing the distribution of TA systems from different strains. Figure S4: Circos plot showing the distribution of structurally validated TA systems from different strains. Supplementary File S1: Annotation of isolates and additional metadata. Supplementary File S2: List of *Campylobacter* reference genomes used for genome annotation. Supplementary File S3: An annotated file for all predicted TA modules from TADB and PADS Arsenal in this study. Supplementary File S4: Predicted type I antitoxins from TADB. Supplementary File S5: Statistical association between the clonal complex and TA family type.
