# Supplementary figures and images for: In Silico Characterization of Toxin-Antitoxin Systems in Campylobacter Isolates Recovered from Food Sources and Sporadic Human Illness
Source: Genes (Basel). 2021 Jan 7;12(1):72. doi: 10.3390/genes12010072 (PMC7826846; doi:10.3390/genes12010072)

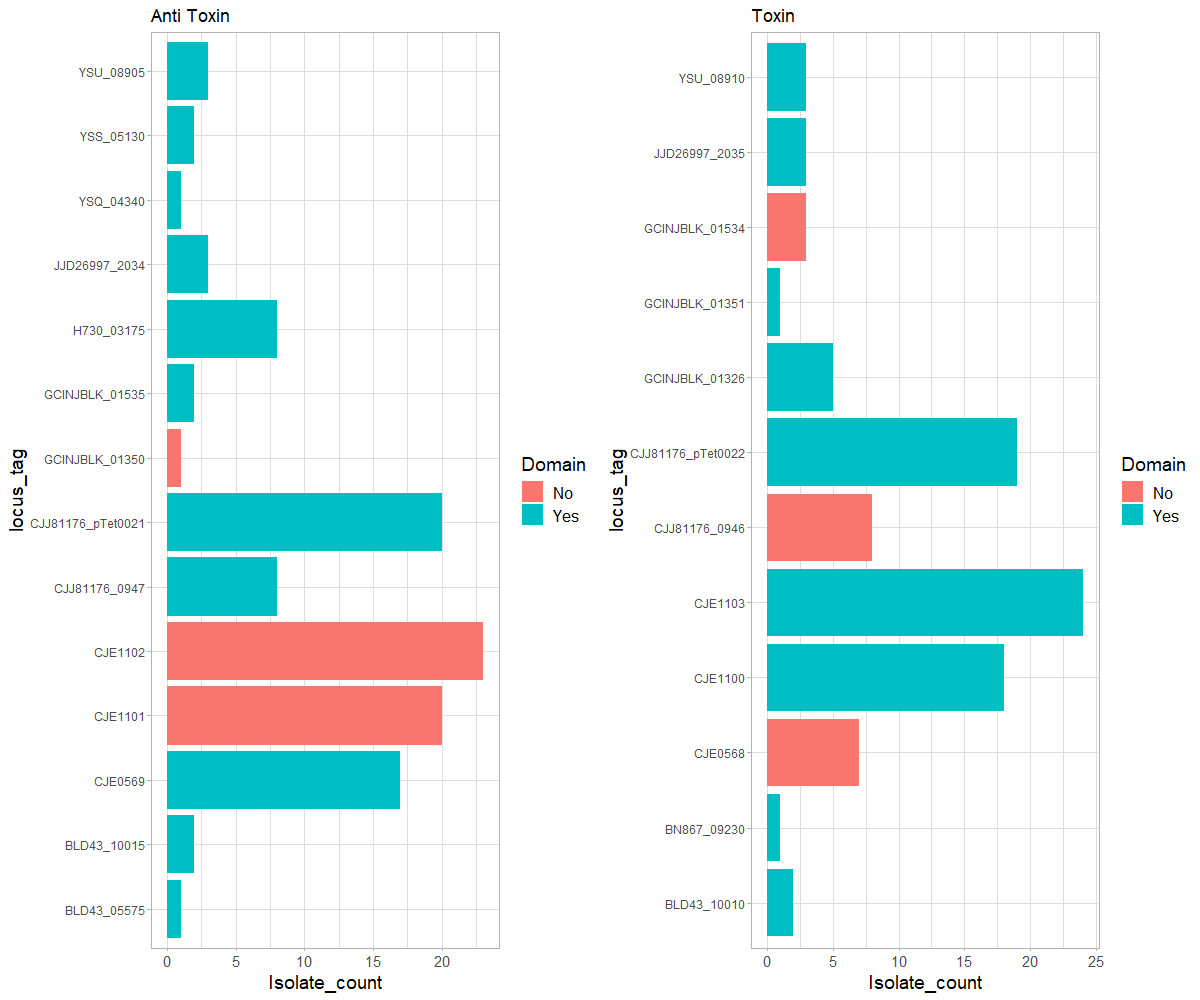

Supplement: Supplementary file 1 [file genes-12-00072-s001.zip › Fig S1.jpeg]

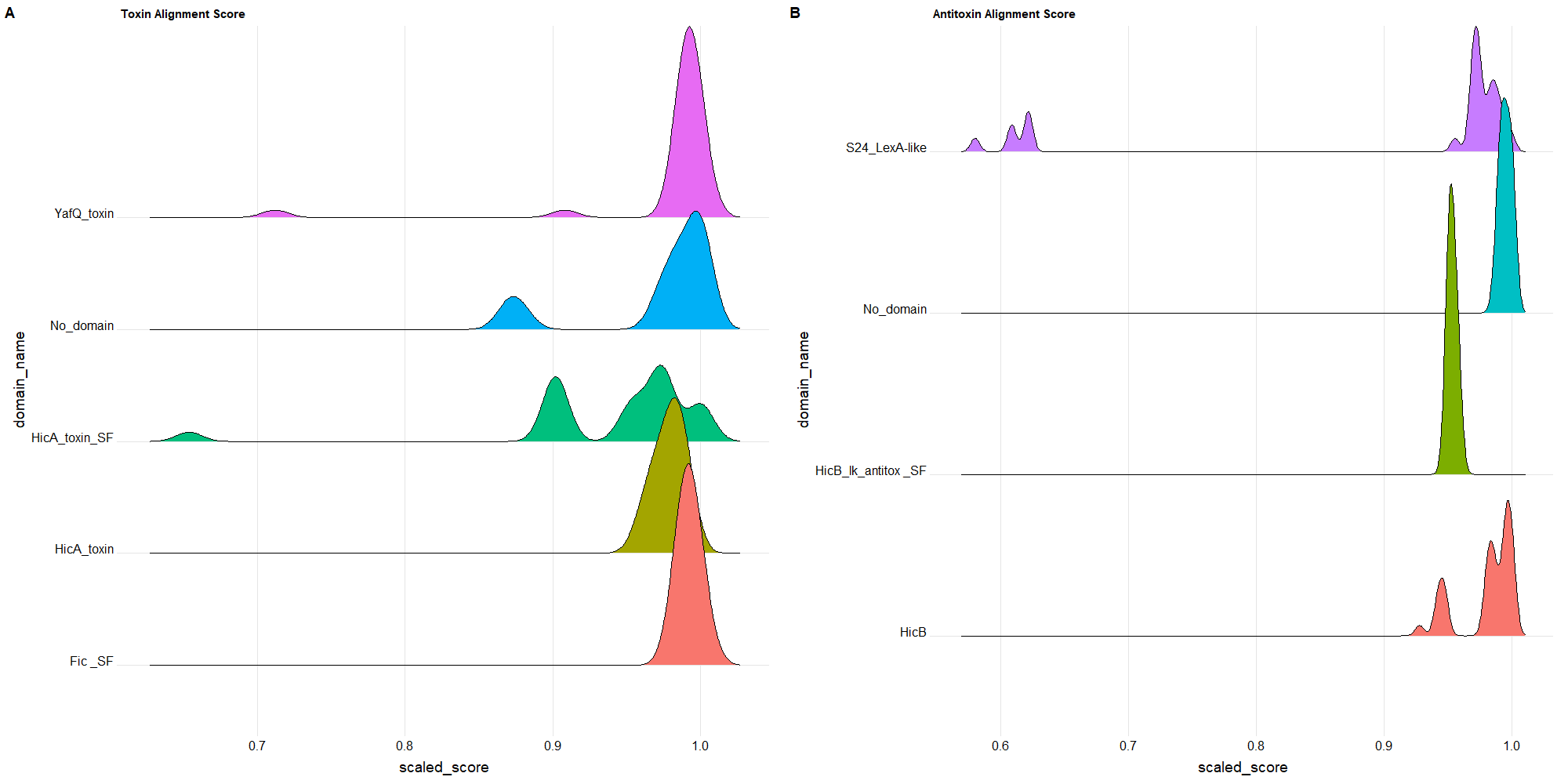

Supplement: Supplementary file 1 [file genes-12-00072-s001.zip › Fig S2.png]

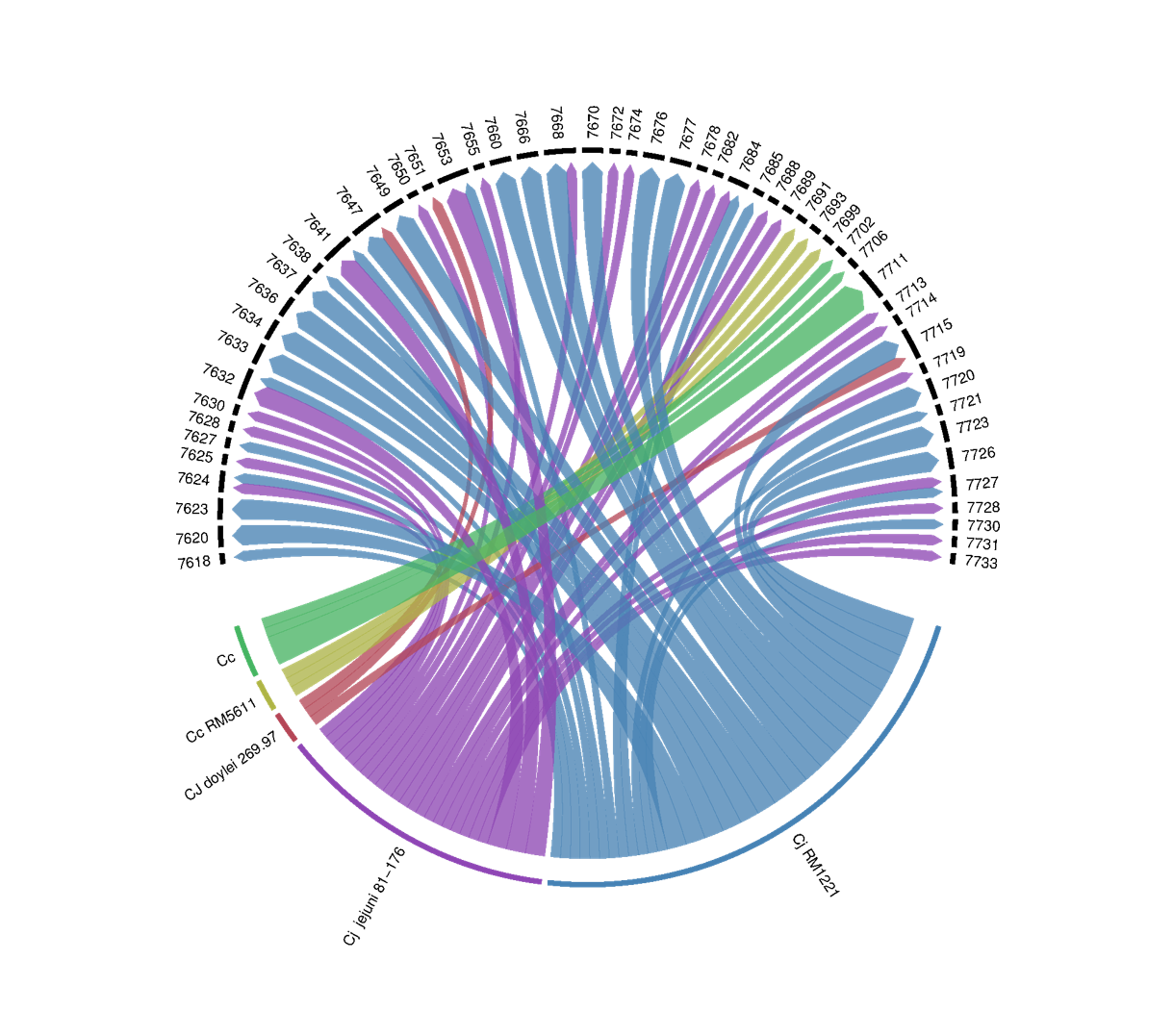

Supplement: Supplementary file 1 [file genes-12-00072-s001.zip › Fig S3.png]

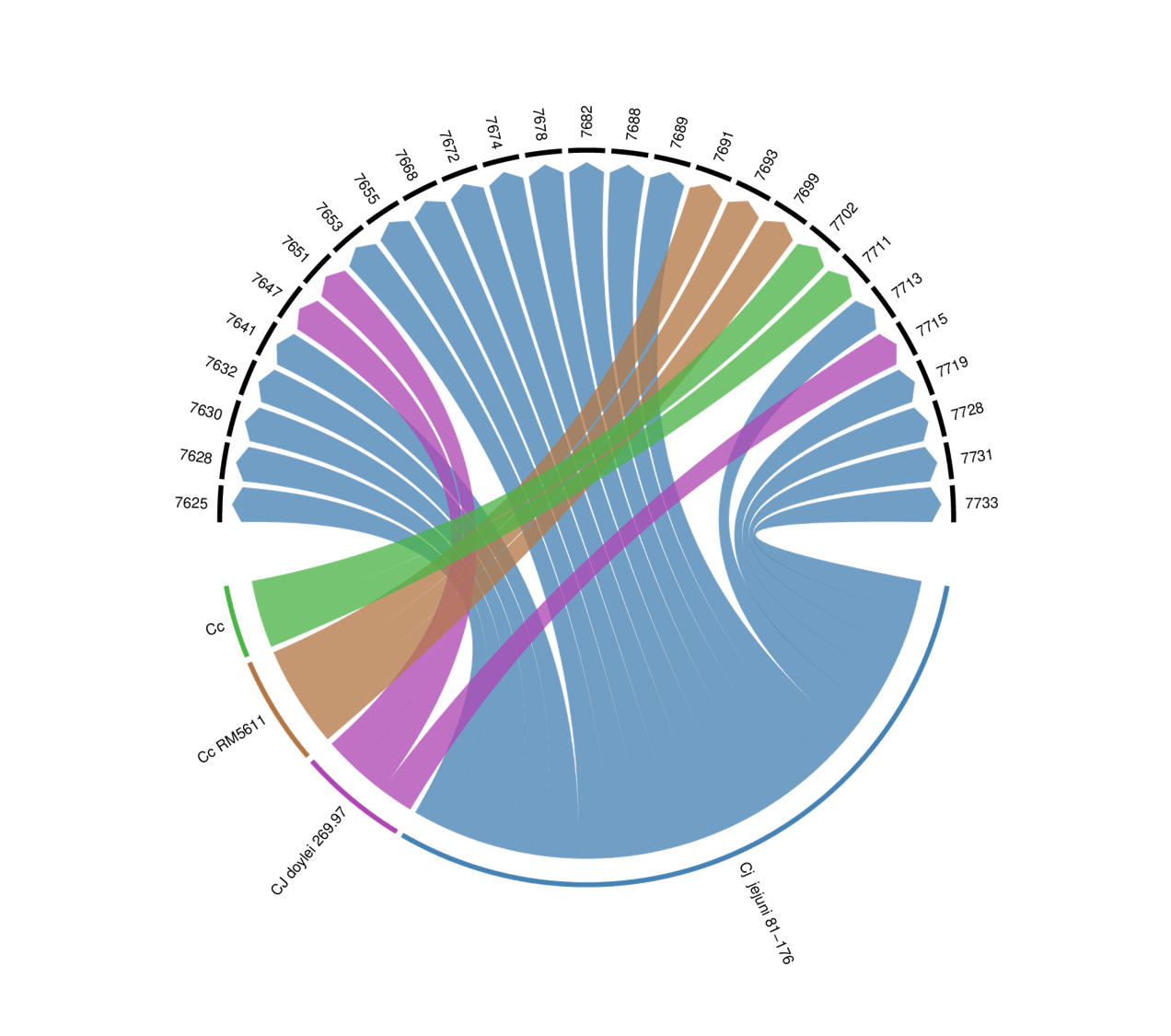

Supplement: Supplementary file 1 [file genes-12-00072-s001.zip › Fig S4.png]
